# Host cellular transcriptional response to respiratory syncytial virus infection in HEp-2 cells: insights from cDNA microarray and quantitative PCR analyses

**DOI:** 10.3389/fcimb.2025.1613386

**Published:** 2025-06-24

**Authors:** Manoj K. Pastey, Christopher Lupfer

**Affiliations:** ^1^ Department of Veterinary Biomedical Sciences, Oregon State University, Corvallis, OR, United States; ^2^ Department of Biology, Missouri State University, Springfield, MO, United States

**Keywords:** respiratory syncytial virus, host-virus interactions, gene expression, host transcriptomic response, microarray

## Abstract

Respiratory syncytial virus (RSV) is a leading cause of lower respiratory tract infections in young children and elderly, worldwide and poses significant risks to immunocompromised individuals. To elucidate host-virus interactions at the transcriptional level, we analyzed differential gene expression in HEp-2 cells infected with RSV using cDNA microarray analysis complemented by quantitative PCR (qPCR). HEp-2 cells were infected with RSV at a multiplicity of infection of 1, and total RNA was isolated 24 hours post-infection for gene expression profiling. Radiolabeled cDNA probes from RSV-infected and mock-infected cells were hybridized to Atlas^®^ Human Cancer cDNA arrays, and differential gene expression was quantified by densitometry. We identified 12 host genes that were significantly upregulated in RSV-infected cells from the cDNA microarray (≥2-fold increase, *P*<0.01), confirmed by qPCR, encompassing functional categories including cell cycle regulation, cytoskeletal organization, apoptosis modulation, immune evasion, and inflammation. Notably, the cyclin-dependent kinase inhibitor CDKN1A was induced ~14-fold, suggesting RSV triggers a host cell cycle arrest. The intermediate filament protein, vimentin was up ~6-fold, consistent with cytoskeletal rearrangements observed during viral syncytium formation. Anti-apoptotic MCL1 increased ~11-fold, while pro-apoptotic caspase-4 showed a more modest 1.6-fold rise, indicating a complex regulation of cell death pathways. We also observed marked upregulation of a fibronectin receptor subunit (~24-fold) and complement regulatory protein CD59 (~2-fold), highlighting potential mechanisms of enhanced cell-cell fusion and viral immune evasion. The proinflammatory cytokine interleukin-6 was elevated ~7-fold, underscoring the inflammatory response to RSV. These findings provide a global snapshot of the host transcriptomic response to RSV infection and yield insights into how RSV modulates host cellular machinery to favor viral replication and spread. Understanding these host-virus interactions may unveil novel targets for antiviral therapy and inform strategies to mitigate RSV disease pathogenesis.

## Introduction

Respiratory syncytial virus (RSV) is the most common cause of severe lower respiratory tract illness in infants and young children, accounting for millions of hospitalizations and a substantial global disease burden (Nair et al., 2010; WHO, 2025). In addition to bronchiolitis and pneumonia in otherwise healthy infants, RSV infection can be life-threatening in certain high-risk groups, including premature infants, the elderly, and patients with compromised immune systems (Koval and Gonzalez, 2024; Whimbey et al., 1995). RSV bronchiolitis in early life is also epidemiologically linked to an increased risk of wheezing and asthma later in childhood (Sigurs et al., 2000), either by causing lasting airway alterations or unmasking an underlying predisposition (a relationship investigated in longitudinal cohort studies) (Sigurs et al., 2000).

At the cellular level, RSV primarily targets airway epithelial cells, which play a pivotal role in initiating the host’s immune response to infection. Infected airway epithelial cells produce a broad array of cytokines and chemokines that orchestrate inflammatory cell recruitment to the lungs (Noah et al., 1995; Morrison et al., 2007). This virus-induced immune response contributes to the pathogenesis of RSV bronchiolitis: accumulation of mucus, fluid, and cellular debris can lead to airway obstruction and impaired gas exchange in severe cases (Morrison et al., 2007). Thus, the clinical outcome of RSV infection reflects a delicate balance between antiviral defense and immunopathology.

Host-virus interactions involve a complex interplay of molecular pathways. While the host cell activates antiviral programs to suppress viral replication, RSV has evolved countermeasures to manipulate host cell processes and evade immune elimination. A powerful approach to unravel these interactions is to profile host gene expression changes during infection. By identifying which host genes are up- or down-regulated in response to RSV, we can infer the pathways hijacked by the virus or mobilized by the host’s defense. Previous studies have shown that many viruses (e.g., HIV-1) actively modulate host cell cycle progression and apoptosis to create a cellular environment conducive to viral replication (Goh et al., 1998). Likewise, RSV is known to induce cell fusion (syncytium formation) and cytoskeletal rearrangements in infected cells, and to interfere with innate immune signaling, though the underlying molecular mediators in host cells are not fully defined (Oshansky et al., 2009).

In this study, we applied cDNA microarray analysis to systematically profile host gene expression in RSV-infected epithelial cells. HEp-2 cells (a human laryngeal carcinoma cell line permissive to RSV) were used as an *in vitro* model of RSV infection. By comparing the transcriptomic profiles of RSV-infected versus mock-infected cells, we identified key host genes and pathways altered by RSV at 24 hpi. We focus on a set of genes that showed significant upregulation upon infection and discuss their potential roles in the virus life cycle and pathogenesis of RSV disease. These data provide insights into the molecular basis of host antiviral responses and viral counterstrategies, laying a foundation for future investigations into therapeutic interventions targeting host-pathogen interactions.

## Materials and methods

### Cell culture and virus infection

HEp-2 cells (ATCC CCL-23) were maintained in Dulbecco’s Modified Eagle Medium (DMEM) supplemented with 10% fetal bovine serum and penicillin-streptomycin at 37°C in a 5% CO_2_ atmosphere. For infection experiments, cells were seeded in six-well plates and grown to ~80% confluence. The cells were then inoculated with RSV (A2 strain) at a multiplicity of infection (MOI) of 1.0 in serum-free DMEM. After 2 hours of adsorption, the inoculum was removed, cells were washed with phosphate-buffered saline (PBS), and fresh growth medium was added. Mock-infected control cells underwent the same procedure with conditioned medium lacking virus. Infected and mock control cultures were incubated for 24 hours post-infection, a time point at which cytopathic effects (such as cell rounding and syncytia formation) were beginning to be observed microscopically.

### RNA isolation and probe preparation

At 24 hpi, cells were harvested for RNA extraction. Total cellular RNA was isolated using the Qiagen RNeasy kit according to the manufacturer’s instructions, including on-column DNase I treatment to remove any genomic DNA. RNA quantity and integrity were verified by UV spectrophotometry and agarose gel electrophoresis. High-quality total RNA (2 μg per sample) was used to synthesize radiolabeled cDNA probes. We employed the Atlas^®^ Pure Total RNA Labeling System (Clontech, Cat. No. 634562) to generate ^32^P-labeled cDNA from each RNA sample via reverse transcription. The RSV-infected and mock-infected cDNA probes were labeled with equal efficiency and purified from unincorporated radionucleotides.

### Microarray hybridization

**Figure 1:**
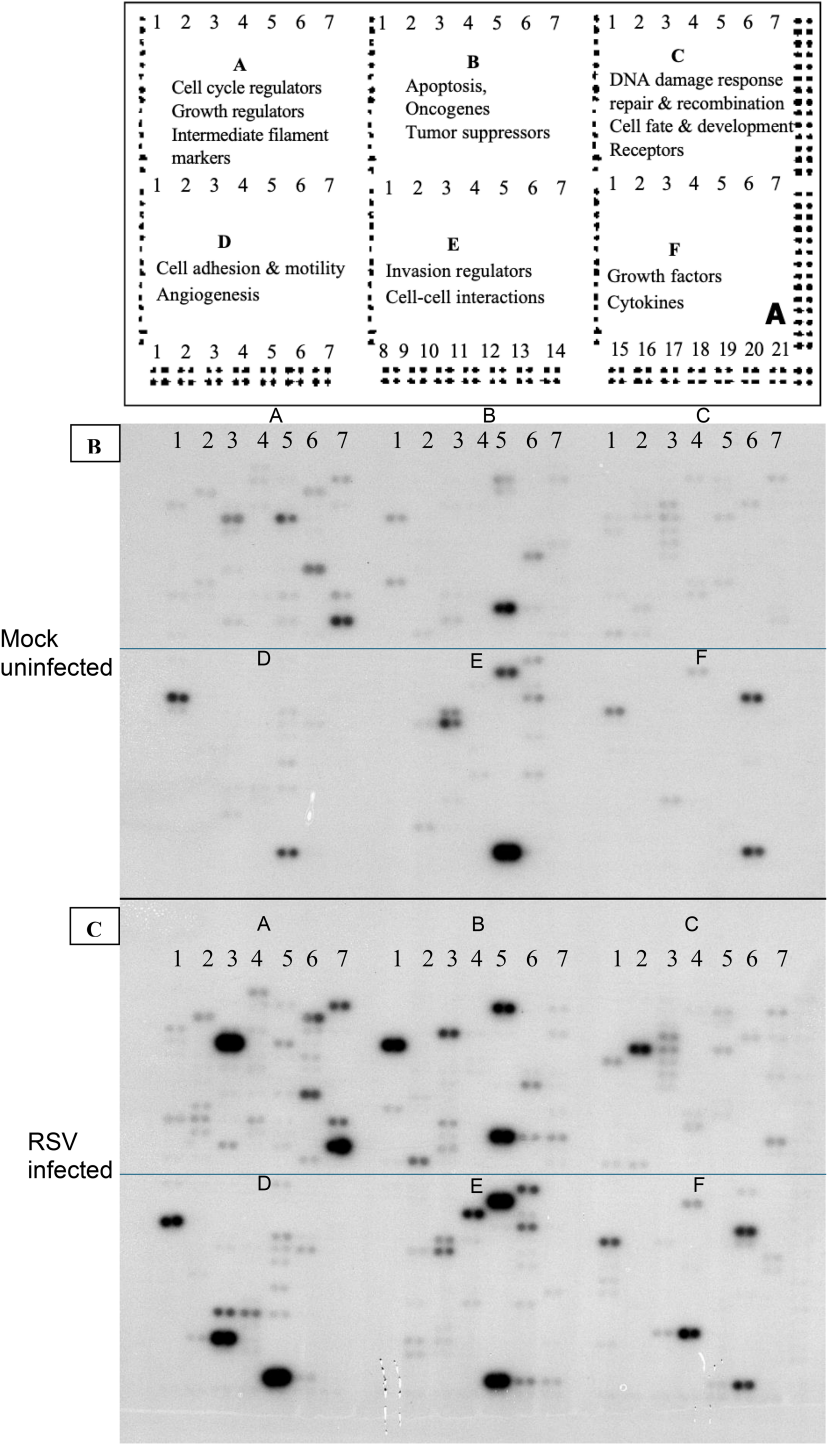
Gene expression profiling using total RNA from RSV or mock-infected cells. The schematic diagram of Atlas human cancer expression array contains 588 cancer-related human cDNA spotted as duplicates. Nine housekeeping genes are spotted at the bottom line to serve as positive controls. Dark grey spots at the outer end of the array represent genomic DNA spots, which serve as orientation marks **(A)**. P^32^-labeled cDNA probes were prepared from 2 μg of total RNA from mock-infected HEp-2 cells **(B)** and RSV-infected HEp-2 cells **(C)** respectively. The probes were hybridized to separate Atlas Human Cancer cDNA Expression Array membranes. Results were analyzed by autoradiography.

Atlas^®^ Human Cancer cDNA Expression Arrays (Clontech, Cat. No. 634511) were used to profile gene expression. These nylon membrane arrays contain 588 spotted cDNA targets (representing a broad panel of human genes, including housekeeping controls). Each array membrane was pre-hybridized in ExpressHyb solution (Clontech) to block non-specific binding, then hybridized overnight at 68°C with either the RSV-derived or mock-derived ^32^P-labeled cDNA probe (each membrane receiving one probe, in parallel). After hybridization, membranes were washed at high stringency (0.1× SSC, 0.5% SDS, at 68°C) to remove non-specifically bound probe. **Figure 1** illustrates the hybridization results: image A shows the schematic diagram of Atlas human cancer expression array containing 588 cancer-related human cDNA spotted as duplicates. Image B shows the autoradiogram of the array probed with mock-infected cell cDNA, and image C shows the array probed with RSV-infected cell cDNA. Each gene is spotted in duplicate on the array, allowing internal confirmation of signal changes.

### Data acquisition and analysis

Hybridized arrays were exposed to Kodak BioMax MS autoradiography film with an intensifying screen at –70°C for 24 hours to visualize the radioactive signals (spots corresponding to expressed genes). The developed autoradiographs were scanned and digitized for analysis. Spot intensities on the arrays were quantified by densitometry using ImageJ (NIH) software. Local background was subtracted, and the average of duplicate spots for each gene was calculated. To identify genes differentially expressed due to RSV infection, we compared the signal intensity for each gene in the RSV-infected sample to the corresponding signal in the mock-infected sample. The ratio (fold change) of expression (RSV/mock) was computed for each gene. For normalization, we initially considered housekeeping genes on the array (such as β-actin, GAPDH); however, one of the polyubiquitin transcripts (Ubiquitin C) traditionally used as a control was itself markedly induced by RSV (see Results), so global normalization was applied using the mean signal of all genes after excluding external spiking controls.

Statistical analysis was performed to determine significant changes in gene expression. We imported the intensity data into S-PLUS 7.0 (Insightful Corp.) for analysis. For each gene, we constructed a confidence interval for the fold-change based on replicate spots and background noise. Genes were considered significantly up- or down-regulated if they exhibited at least a two-fold change in expression in RSV-infected cells relative to mock-infected controls, with statistical significance at the 99% confidence interval (approximately P < 0.01). This stringent threshold was intentionally chosen to identify robust and biologically meaningful transcriptional alterations while minimizing false positives resulting from background variability or technical noise inherent to array-based assays. As a result, the analysis yielded a focused set of genes with major expression changes, which are likely to be functionally relevant in the context of RSV infection. These significantly altered genes are summarized in **Table 1** for further interpretation.

**Table 1:** Significantly upregulated host genes in RSV-infected HEp-2 cells (24 hpi).

Functional Category^a^	**Gene Symbol**	**GeneBank**	Blot Position^b^	Fold Change^c^ **(RSV/Mock)**
Stress Response	UBC	M26880	G5	13.64 ↑
	GADD153	S40706	C2e	3.44 ↑
	GSTTLp28	U90313	B5b	4.50 ↑
Cytoskeleton & Motility	TUBA1	K00558	G13	0.31 ↑
	ACTB	X00351	G19	0.06 ↑
	VIM	X56134	A7m	6.37 ↑
	KRT18	M26326	A7b	0.84 ↑
Oncogenes & Tumor Suppressors	RHOA	L25080	B5l	3.67 ↑
	RHO8	X95282	E4c	3.52 ↑
	CDKN1A	U09579	A3e	14.26 ↑
	RHO-GDI1	X69550	E5b	8.97 ↑
Cell Adhesion & Signaling	ITGA3	M59911	D3k	0.82 ↑
	FNRA	X06256	D3m	24.13 ↑
	CTNNA1	D13866	E6a	0.84 ↑
Immune & Antigen Presentation	HLAC	M11886	G14	0.13 ↑
	IL6	X04602	F4l	6.83 ↑
	CD59	M34671	D1d	2.06 ↑
Apoptosis	CASP4	U28014	B3d	1.63 ↑
	MCL1	L08246	B1e	11.19↑

Fold change represents the ratio of expression in RSV-infected vs. mock-infected cells, and functional categories are based on known gene roles.

a Genes grouped by their known functional roles based on significant transcriptional changes observed in RSV-infected cells.

b Blot Position refers to specific array coordinates used during microarray analysis.

c Fold Change: ↑ indicates upregulation; values represent fold change in RSV-infected cells relative to mock-infected controls with 99% confidence in microarray analysis.

**Figure 2:**
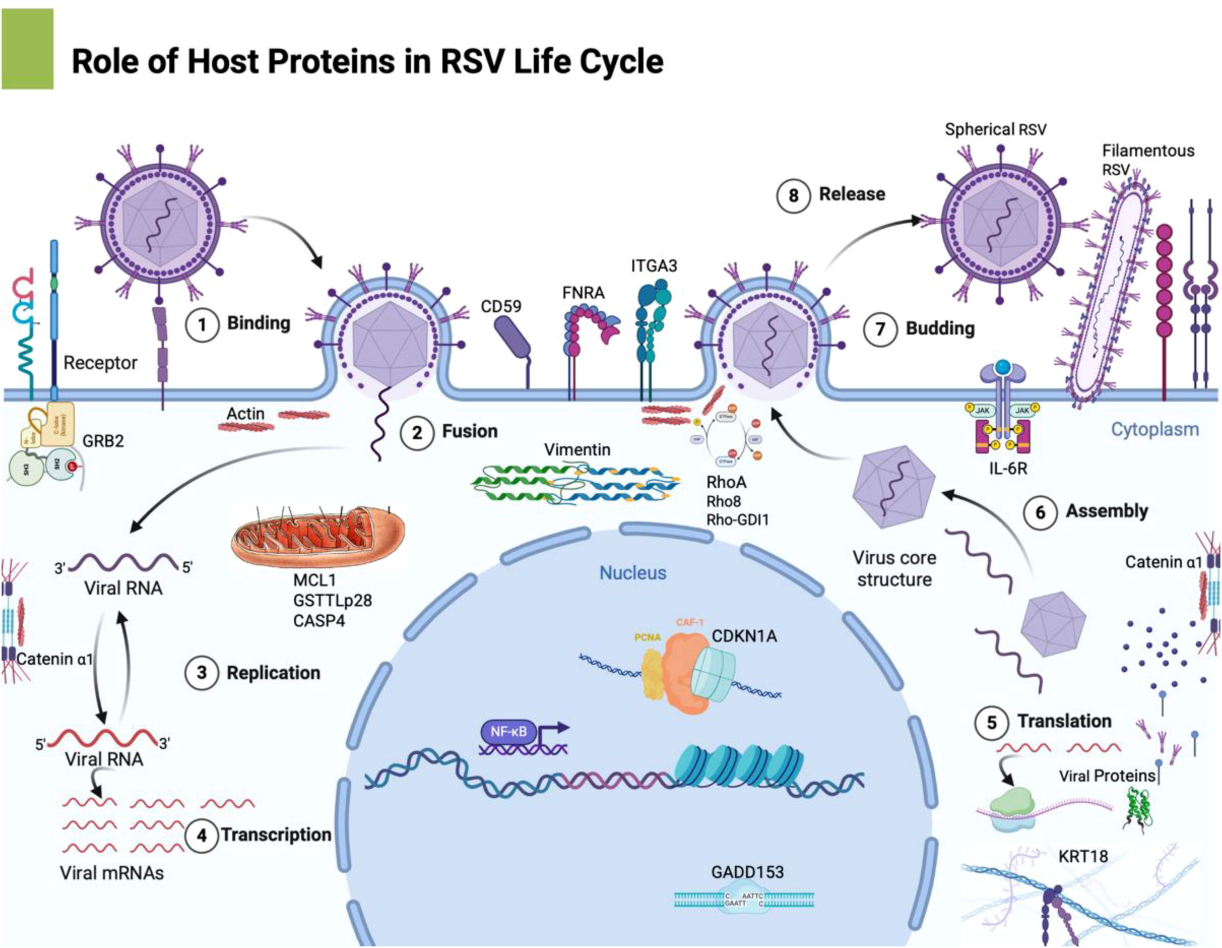
This schematic representation outlines the major host pathways disrupted by RSV infection: This refined schematic visually summarizes how RSV infection orchestrates changes in host cell functions, highlighting key pathways that contribute to its pathogenesis and potential targets for therapeutic intervention. Image was created with Biorender software. 1. Cell Cycle Dysregulation: RSV upregulates CDKN1A (p21), leading to G1 phase arrest, which prevents normal cell cycle progression, potentially benefiting viral replication. 2. Cytoskeletal Reorganization: The upregulation of vimentin alters intermediate filaments, contributing to syncytium formation—a hallmark of RSV infection that facilitates viral spread. 3. Apoptosis Modulation: RSV increases MCL1 expression and modulates caspase 4 activity, delaying apoptosis and extending the period of viral replication within the host cell. 4. Cell Adhesion & Immune Evasion: The upregulation of FNRA enhances cell-matrix adhesion, while CD59 expression protects infected cells from complement-mediated lysis, helping the virus evade immune clearance. 5.Inflammatory Response: RSV significantly increases pro-inflammatory cytokines like IL-6 and TNF-α, leading to excessive immune activation, which contributes to tissue damage and pathogenesis.

Additionally, to validate the microarray findings, we conducted quantitative real-time PCR (qPCR) on a subset of the differentially expressed genes. cDNA was prepared from independent RNA isolates of mock and RSV-infected HEp-2 cells (24 hpi), and qPCR was performed using gene-specific primers (designed for CDKN1A, IL6, MCL1, and others) designed using NCBI Primer-BLAST, with SYBR Green detection on an ABI Prism system. Expression levels were normalized to an internal reference (18S rRNA) and calculated by the delta-delta CT (ΔΔC_t) method. The qPCR results were compared to the microarray fold-change data for consistency. PCR primer sequences and cycle threshold (Ct) values are provided in **Table 2**. We have provided a refined schematic figure which visually summarizes how RSV infection orchestrates changes in host cell functions, highlighting key pathways that contribute to its pathogenesis and potential targets for therapeutic intervention (**Figure 2**).

**Table 2:** Quantitative Real-Time PCR (qPCR) validation of selected differentially expressed genes in RSV-Infected HEp-2 Cells.

Gene^a^	Fold change^b^ RSV/Mock	Ct^c^	Forward Primer (5'→3')^d^	Reverse Primer (5'→3')^d^
UBC	14.14 ↑	21.0	GGAGTTGGCGAGTGTGTTTT	ACGGCCAGAATTTAGCGGAC
PLA2	0.17 ↑	27.6	CGTCCCTCAAACCTTGCTTC	GTTAAGGGCCAGACCCAGTC
GAPDH	-0.40 ↓	25.8	ACGCAAAAGAAGATGCGGCT	TGGAATTTGCCATGGGTGGA
TUBA1	0.39 ↑	26.4	GAGCGCCCAACCTACACTAA	ATCCACAAACTGGATCGTGC
HLAC	0.21 ↑	27.3	AGTATTGGGACCGGGAGACA	GGTATCTGCGGAGCCACTC
ACTB	0.1 ↑	28.3	AGACCTGTACGCCAACACAG	CCTCGGCCACATTGTGAACT
RPL13A	0.35 ↑	26.5	GTGGTCGTACGCTGTGAAGG	TTTTGTGGGGCAGCATACCT
CDKN1A	19.56 ↑	20.3	GTCAGTTCCTTGTGGAGCCG	GAAGGTAGAGCTTGGGCAGG
VIM	8.83 ↑	21.9	GCTACGTGACTACGTCCACC	TAGTTGGCGAAGCGGTCATT
KRT18	0.98 ↑	25.0	CTCTCCCCGGACAGCATGAG	TCTGTCCAGGTAAGAGGCCA
MCL1	13.31 ↑	21.1	TGGTGGGTTTATAGGGGAGGA	TCCTAACCCTTCCTGGCACA
GSTTLp28	5.67 ↑	22.8	AGGAGCTTGGGGAAGGGAAG	TTCCAGAACTGGCACCAGAC
RHOA	6.22 ↑	22.4	CTGGGGTGGGCAGTTTTGA	GAGCCAGACGCTTAAGTCC
CASP4	2.37 ↑	24.3	TGTTCCCTATGGCAGAAGGC	GGTCCAGCCTCCATATTCGG
GADD153	4.20 ↑	23.3	ATTGCCTTTCTCTTCGGACAC	TCTGGAGAGTGAGGGCTCTG
CD59	2.39 ↑	24.3	CCGCCAGGTTCTGTGGAC	TTTTCCCTCAAGCGGGTTGT
FNRA	27.43 ↑	19.2	GACAGATGCCACAAGGATAG	TGCCAATGTCTGAGTCTGGG
ITGA3	1.42 ↑	24.7	GGATCCATCTTGAGAGCCACA	GTTGCCGTTTGCCTGTCTTT
CTNNA1	1.22 ↑	24.9	CCTCTGGAATTTAGCGCTCG	AGAAACTGGCTCTCCTTCGC
RhoE	4.15 ↑	23.3	TGGGAGACAGTCAGTGTGGA	CACAGCATCCGAATCAGGGT
RHO-GDI1	9.74 ↑	21.8	CTGGACAAGGACGACGAGAG	GTCCGTCTTCCGTCCATCAC
IL6	7.93 ↑	22.0	TTCGGTCCAGTTGCCTTCTC	CAGCTCTGGCTTGTTCCTCA
GRB2	0.77 ↑	25.4	CCGTGAACCGGAACGTCTAA	AGCTAGGACTATACGTGGCCT

a Genes that showed significant transcriptional changes observed in RSV-infected cells.

b Fold Changes marked with arrows (↑, upregulated; ↓, downregulated); values represent fold change in RSV-infected cells relative to mock-infected controls with 99% confidence.

c PCR cycle threshold (Ct) values. Lower Ct values indicate higher gene expression. Expression levels were normalized to 18S rRNA and calculated using the delta-delta Ct (ΔΔC_t) method. The qPCR results were compared to the microarray fold-change data for consistency.

dPCR gene-specific primer sequences designed using NCBI Primer-BLAST are provided.

## Results

### Global gene expression changes induced by RSV

The cDNA microarray analysis revealed that RSV infection triggers broad transcriptional alterations in HEp-2 cells by 24 hours post-infection. Hybridization patterns on the array were distinct between RSV-infected and mock-infected samples (**Figure 1**), indicating differential expression of multiple genes. Using a cutoff of ≥2.0-fold change at 99% confidence, we identified 12 host genes that were significantly upregulated in RSV-infected cells compared to controls (**Table 1**). No transcripts met these strict criteria for significant downregulation, suggesting that the early host response to RSV is dominated by inducive (upregulatory) events, at least among the genes represented on this array. The list of upregulated genes, their approximate fold increases, and their functional classifications are summarized in **Table 1**. Quantitative PCR validation for selected genes (including CDKN1A, IL6, MCL1) supported the direction and relative magnitude of these changes, as RSV-infected samples showed consistently lower C_t_ values (indicative of higher mRNA levels) than mock samples for these targets (**Table 2**). **Figure 2** shows a schematic representation outlining the major host pathways disrupted by RSV infection.

### Cell cycle arrest gene upregulation

Among the most strongly induced transcripts was cyclin-dependent kinase inhibitor 1A (CDKN1A), also known as *p^21^Cip1/Waf1*. CDKN1A mRNA levels increased by approximately 14-fold in RSV-infected cells (**Table 1**). *p21* is a well-known mediator of cell cycle G1 phase arrest, typically under the control of the tumor suppressor p53. Its upregulation suggests that RSV infection may activate a p53–p21 pathway to halt cell cycle progression. Functionally, a virus-induced G1 arrest can be a double-edged sword: it may represent a host defense to prevent virus spread, or conversely, a viral strategy to create a more favorable environment for replication. Similar observations have been made with other viruses (for instance, HIV-1 Vpr protein causes G2/M cell cycle arrest to enhance viral yield (Goh et al., 1998). In our system, the robust induction of p21 hints that RSV triggers a DNA damage or stress response in epithelial cells leading to cell cycle checkpoint activation.

### Cytoskeletal and adhesion molecules

The microarray data indicated significant remodeling of cytoskeletal component expression. Vimentin, a type III intermediate filament protein, was upregulated ~6.4-fold by RSV. Vimentin is part of the host cell scaffolding that maintains cell shape and organelle position, and it is notably involved in the formation of virus-induced syncytia (multinucleated giant cells) in RSV infection (Garcia-Barreno et al., 1988). The increase in vimentin expression aligns with known RSV cytopathic effects: infected HEp-2 cells tend to form syncytia, and prior studies have reported redistribution and even proteolytic cleavage of intermediate filaments during RSV infection (Garcia-Barreno et al., 1988). Here, the elevated vimentin transcript suggests an attempted compensatory response to cytoskeletal stress or a viral induction to facilitate syncytium formation. Another intermediate filament gene, keratin 18 (KRT18), showed a slight increase (~1.8-fold by densitometry, not reaching our significance threshold). While not statistically significant, this trend along with vimentin’s strong induction underscores that RSV impacts the intermediate filament network of the host cell.

Changes were also observed in genes related to cell adhesion and the extracellular matrix. The fibronectin receptor alpha subunit (FNRA), which corresponds to integrin α5 (part of the α5β1 fibronectin receptor), was the most highly induced gene on the array, with a ~24-fold increase in expression. Integrin α5β1 is crucial for binding fibronectin and assembling extracellular matrix fibrils (Bachmann et al., 2019). Upregulation of FNRA may facilitate cell-cell attachment and the extensive membrane fusion characteristic of RSV infection. Indeed, integrin engagement can activate RhoA signaling pathways (Danen et al., 2002), and as discussed below, RhoA is required for efficient RSV syncytium formation (Gower et al., 2005). We also noted modest increases in integrin α3 (ITGA3) and α-catenin (CTNNA1) transcripts (approximately 1.8-fold each, albeit below the cut-off for high confidence). Integrin α3β1 is a receptor for laminin involved in cell-matrix adhesion and has been implicated in cell signaling and possibly gap junction communication (Bachmann et al., 2019). α-catenin links cadherin adhesion complexes to the actin cytoskeleton. Although the changes in ITGA3 and CTNNA1 were not large enough to be deemed significant in this dataset, their slight elevation might contribute to subtle modifications in cell-cell contacts during infection. Upregulation of adhesion molecules like integrins and catenins could support the extensive cell clustering and fusion caused by RSV.

### Apoptosis regulatory genes

RSV-infected HEp-2 cells showed differential expression of genes involved in apoptosis (programmed cell death). MCL1, an anti-apoptotic member of the Bcl-2 protein family, was strongly upregulated (~11.2-fold). MCL1 protein is known to antagonize the mitochondrial (intrinsic) apoptosis pathway and prolong cell survival. Its induction by RSV suggests that infected cells may delay the onset of apoptosis, potentially allowing the virus more time to replicate and spread. In contrast, we observed a smaller increase in CASP4 (caspase-4) mRNA (~1.6-fold). Caspase-4 is an inflammatory caspase (related to caspase-1 subfamily) and can mediate a form of cell death associated with endoplasmic reticulum stress. Although the caspase-4 change did not meet the 2-fold threshold, it is notable because prior studies have linked RSV-induced syncytium formation to apoptosis in the later stages of infection, possibly via intrinsic pathways (Oshansky et al., 2009). The concurrent upregulation of an anti-apoptotic factor (MCL1) with only a mild rise in a pro-apoptotic caspase suggests that RSV tilts the balance toward cell survival in the early phase of infection. Such a strategy – holding off host cell death – is common among viruses to maximize production of progeny. Indeed, other viruses like HIV-1 have been reported to delay apoptosis early (e.g., through Vpr or Nef proteins) and then trigger cell death at a time that may facilitate viral dissemination (Mbita et al., 2014; Stewart et al., 2000). Our data imply RSV might employ a similar tactic: MCL1 induction could inhibit premature apoptosis (Oshansky et al., 2009), thereby extending the lifespan of the infected cell until viral assembly and egress are accomplished.

Another gene in the apoptosis/DNA damage category, GADD153 (also known as CHOP, a transcription factor activated by ER stress and DNA damage), was elevated ~3.5-fold. CHOP is generally pro-apoptotic and is induced by stresses such as unfolded protein response; its upregulation can synergize with cytokine production, for instance by enhancing IL-6 expression under certain conditions (Smith, 2018). The increase in GADD153 could indicate RSV imposes enough stress on the ER (perhaps through high levels of viral protein synthesis) to activate this pathway, which might eventually contribute to cell death and inflammatory signaling.

### Stress and metabolism-related genes

The GSTTLp28 gene (glutathione S-transferase theta-like protein) showed a 4.5-fold increase. This gene is related to the glutathione S-transferase family, enzymes involved in detoxification of reactive oxygen species (ROS) and toxic compounds by conjugation to glutathione (Townsend and Tew, 2003). RSV infection is known to induce oxidative stress and an inflammatory milieu in the airway (Yang et al., 2023). Induction of a GST enzyme in infected cells likely represents a host protective response, aiming to neutralize ROS generated either by cell metabolic changes or by immune cell-produced oxidants. By boosting GST levels, the cell may mitigate oxidative damage during infection (Hosakote et al., 2009). This aligns with reports that antioxidants can modulate RSV-induced cytokine production, highlighting the role of oxidative stress in RSV pathogenesis (Hosakote et al., 2009).

We also observed a striking change in a gene that was *intended* as an internal control: Ubiquitin C (UBC). UBC encodes a polyubiquitin precursor (multiple repeats of ubiquitin protein) and is usually constitutively expressed for protein degradation needs. Interestingly, in RSV-infected cells UBC transcript levels were ~13.6-fold higher than in controls. This unexpected surge suggests that RSV infection triggers a strong activation of the ubiquitin-proteasome system. It is possible that the accumulation of misfolded proteins or the need to degrade certain host or viral proteins during infection drives up ubiquitin expression. Alternatively, RSV may actively manipulate ubiquitination pathways as an immune evasion strategy (for instance, some viral proteins co-opt ubiquitin ligase complexes to target antiviral signaling molecules for degradation). The upregulation of UBC cautioned us against using it (or similar “housekeeping” genes) as a normalization reference in our array analysis. Instead, it became an important finding on its own, hinting at involvement of ubiquitin-mediated processes in RSV infection (explored further in the Discussion).

### Immune and inflammatory response genes

Components of the immune response were also among the upregulated genes. Interleukin-6 (IL-6), a multifunctional cytokine, was significantly increased (~6.8-fold). IL-6 is produced by airway epithelial cells and various immune cells in response to viral infections and tissue damage. In the context of RSV, IL-6 has a dual role: it contributes to the acute inflammatory response (fever, acute phase protein production, leukocyte recruitment) and also has immunoregulatory effects (e.g., promoting B-cell maturation and T-cell activation) (Castell et al., 1989; Maeda et al., 2010). Elevated IL-6 in RSV-infected cells is consistent with clinical observations that IL-6 levels are detectable in respiratory secretions of infants with RSV bronchiolitis (Levitz et al., 2012). High concentrations of IL-6 in the airways have been associated with severe RSV disease, such as those requiring mechanical ventilation (Russell et al., 2017). Our *in vitro* finding that RSV directly induces IL-6 in epithelial cells supports the idea that the airway epithelium is a key source of cytokines that drive the inflammation seen in RSV infection. The microarray also included other cytokines/chemokines (e.g., IL-8, RANTES/CCL5), but IL-6 was the one markedly elevated at 24 hpi in this system, highlighting its prominence in the early response.

Another gene of note is CD59, which was up ~2.1-fold. CD59 is a membrane-bound glycoprotein that protects host cells from complement-mediated lysis by inhibiting the formation of the membrane attack complex (MAC). Many viruses, especially enveloped viruses, have evolved to exploit host cell membranes and incorporate regulatory proteins like CD59 into their viral envelope as they bud out of cells (Favoreel et al., 2003; Agrawal et al., 2017). The modest increase in CD59 expression could be part of a host attempt to safeguard infected cells from immune destruction. However, it might actually benefit RSV: by acquiring more CD59 on the cell surface (and subsequently on released virions), the virus could evade complement attack in the extracellular environment. This phenomenon has been documented in other viruses (e.g., HIV-1, cytomegalovirus incorporate CD55/CD59 to resist complement (Agrawal et al., 2017). In the case of RSV, there is evidence that the virus associates with lipid raft microdomains rich in complement regulators and incorporates CD55 and CD59 into its envelope (Brown et al., 2004). Thus, upregulation of CD59 in RSV-infected cells may facilitate immune evasion by the virus, ensuring that newly formed virions and infected cells are less susceptible to complement-mediated destruction.

### Confirmation by qPCR

As noted, we performed qPCR validation on all the 23 genes to confirm the microarray results. The qPCR data were in qualitative agreement with the array findings: RSV-infected samples showed substantial increases in p21 and IL-6 mRNA levels, and a pronounced induction of MCL1, relative to mock-infected controls. FNRA mRNA was also confirmed to be dramatically elevated in infected cells. The consistency between qPCR and microarray lends confidence that the observed gene expression changes are genuine and reproducible.

Microarray analysis identified 12 significantly upregulated genes (≥2-fold, p<0.01) at 24 hpi, validated by qPCR. CDKN1A showed a dramatic 14-fold induction on microarray analysis (qPCR Ct 19.56), suggesting RSV-induced cell cycle arrest. Cytoskeletal reorganization markers like vimentin (6-fold; Ct 21.9) were significantly elevated, correlating with RSV-induced syncytium formation. Anti-apoptotic gene MCL1 (11-fold; Ct 21.1) and apoptosis-associated CASP4 (1.6-fold; Ct 24.3) indicated strategic modulation of apoptosis. CD59 (2-fold; Ct 24.3) and FNRA (24-fold; Ct 19.2) upregulation highlighted mechanisms for immune evasion and enhanced cell adhesion facilitating viral spread. IL-6 was elevated nearly 7-fold (Ct 22.0), reinforcing inflammation’s central role in RSV pathology.

In summary, RSV infection of HEp-2 cells resulted in significant upregulation of a discrete set of host genes by 24 hours post-infection (**Figures 1**, **2**). These genes suggest that RSV triggers host pathways involving cell cycle arrest, cytoskeletal reorganization, modulation of apoptosis, oxidative stress responses, cell adhesion, complement inhibition, and cytokine production (**Table 1**). We did not detect any genes with significant downregulation exceeding two-fold at this time point under our conditions, which may indicate that major transcriptional repression by RSV (e.g., interferon responses) could occur either earlier than 24h or involve genes not present on this array. The focus of our findings is on host factors whose increased expression may influence RSV replication and pathogenesis, as discussed below.

## Discussion

In this study, we used cDNA microarrays to profile the transcriptional response of human epithelial cells to RSV infection, providing a snapshot of host-pathogen interactions at the molecular level. Our findings illustrate that RSV elicits a complex host response, with coordinated changes in genes that govern cell cycle progression, cytoskeletal architecture, apoptotic balance, immune modulation, and inflammatory signaling (**Figures 1**, **2**). We identified 12 genes that were significantly upregulated in RSV-infected HEp-2 cells (**Table 1**), and these changes shed light on how RSV manipulates the host cell environment to its advantage while the host mounts defenses.

Several transcriptomic studies have characterized host gene expression responses to RSV infection, consistently reporting robust activation of immune and inflammatory pathways. Ampuero et al. (2018) identified 27 genes that remained upregulated through 24-, 48-, and 72-hours post-infection, mainly related to innate immune responses, including dsRNA sensors, interferon (IFN) production, IFN-stimulated genes (ISGs), and antigen presentation pathways. Zhang et al. (2001) used cDNA microarrays and oligonucleotide microarrays in A549 cells to reveal strong induction of chemokines such as CC (e.g., RANTES, MCP-1, MIP-1α/β), CXC (e.g., IL-8, GRO-α/β/γ, ENA-78), and CX_3_C (e.g., fractalkine). Tian et al. (2002) further demonstrated NF-κB-dependent gene networks activated during RSV infection. Oshansky et al. (2009) and Surolia and Antony (2022) also reported gene networks involved in immune modulation and cytoskeletal remodeling. In a time-course analysis by Martínez et al. (2007), cDNA microarray profiling in A549 cells revealed a predominantly up-regulatory, temporally structured host response, with early induction of PLAU, PLAUR, and integrins, followed by upregulation of ISG15, MX1, non-canonical NF-κB pathway components, and novel genes such as MCL1 and FNRA.

One of the most striking observations in our study was the robust upregulation of CDKN1A (p^21^Cip1), implicating activation of a cell cycle arrest in infected cells. *p21* is a downstream effector of p53; its induction typically leads to G1 phase arrest and prevents cells from entering S-phase. The RSV-mediated increase in p21 expression suggests that infected cells may undergo a transient growth arrest. This could be a deliberate viral strategy: forcing the host cell out of the cell cycle might divert cellular resources towards viral replication or prevent apoptosis that is linked to cell cycle progression (Granville et al., 1998). Analogous mechanisms are seen in other viral infections—HIV-1, for example, through its Vpr protein, causes accumulation of host cells in G2, which has been shown to enhance HIV replication (Elder et al., 2000). While RSV is very different from HIV, the concept of cell cycle manipulation is conserved. Alternatively, the p21 increase might reflect a host stress response, with the cell attempting to limit proliferation due to DNA damage or other stress signals triggered by RSV infection. Indeed, RSV has been reported to induce DNA damage signaling in airway cells in some studies (Yang et al., 2023). Regardless of cause, the consequence is that RSV-infected cells are likely not dividing, which could be beneficial for the virus by maintaining a stable intracellular environment.

The substantial changes in cytoskeletal-related genes (vimentin, integrins, catenin) highlight the importance of cytoskeletal remodeling in RSV infection. Vimentin acts as a receptor or coreceptor for various viruses, including dengue, influenza A, SARS-CoV, and SARS-CoV-2, facilitating entry and replication (Arrindell and Desnues, 2023). Disruption of vimentin significantly impairs viral genome transport, replication, and egress, highlighting its critical role in viral pathogenesis (Arrindell and Desnues, 2023). RSV is well-known for causing infected cells to fuse together, forming syncytia. This process requires dynamic reorganization of the actin cytoskeleton and intermediate filaments (Garcia-Barreno et al., 1988). Our data show vimentin mRNA is significantly elevated, aligning with reports that RSV infection leads to redistribution and even upregulation of vimentin protein in host cells (Surolia and Antony, 2022; Garcia-Barreno et al., 1988). Vimentin may facilitate the merger of cell membranes during syncytium formation, possibly by interacting with viral proteins or by altering cell stiffness and structure. Interestingly, RhoA, a small GTPase that regulates actin stress fibers, was also upregulated. Prior research demonstrated that RhoA activity is required for RSV-induced syncytium formation and the characteristic filamentous morphology of RSV virions (Gower et al., 2005). Our finding of increased RhoA transcript suggests RSV might stimulate the RhoA pathway to promote the cytoskeletal changes necessary for efficient fusion and viral spread. Consistent with this, we saw an increase in integrin α5 (FNRA), which can activate RhoA signaling when engaged by fibronectin (Danen et al., 2002). We also detected upregulation of RhoGDI1 and RhoE (Rnd3), which are modulators of Rho GTPases; RhoGDI1 sequesters Rho proteins in the cytosol, and RhoE is a RhoA antagonist. The concurrent induction of both RhoA and its regulators RhoGDI and RhoE suggests a nuanced modulation of Rho signaling. It is tempting to speculate that the host cell upregulates RhoGDI1 and RhoE as a counter-regulatory mechanism to avoid excessive RhoA activation and preserve junctional integrity, since uncontrolled RhoA activity can disrupt tight junctions. In summary, the transcriptional changes in cytoskeletal regulators underscore a tug-of-war: RSV pushing the cell towards a state favoring fusion and structural rearrangement, and the cell responding by trying to maintain or restore order.

Our data also indicate that RSV modulates apoptotic pathways in infected cells. The virus appears to create an environment that delays apoptosis, at least in the early stages of infection. The strong upregulation of MCL1 likely contributes to cell survival. MCL1 protein can inhibit key pro-apoptotic factors (such as Bax/Bak) and thereby prevent mitochondrial outer membrane permeabilization, a point of no return in the intrinsic apoptosis pathway. By elevating MCL1, RSV-infected cells might resist premature apoptotic death, allowing prolonged virion production. This interpretation is consistent with the observation that caspase-4 was only mildly upregulated. If RSV were triggering robust apoptosis at 24 h, one might expect higher induction of executioner caspases or other pro-apoptotic genes. Instead, the picture aligns with a pro-survival bias. Previous studies of RSV have noted that the virus often keeps the host cell alive until late in the replication cycle; only at later times (48–72 hpi) do infected cells undergo significant apoptosis, which can aid in virus release and dissemination by breaking down cell structures (Oshansky et al., 2009). The interplay of MCL1 and caspase-4 in our results suggests RSV fine-tunes the apoptotic threshold: enough to eventually kill the cell (and cause inflammatory cell death that might help virus spread or immune cell activation), but not so early that it aborts viral replication. In a broader context, this strategy is reminiscent of persistent viruses or those requiring time to assemble – they often encode or induce anti-apoptotic functions to extend the life of the host cell. While RSV is an acute lytic virus, it still benefits from a brief extension of host cell viability (Bedient et al., 2020).

The upregulation of UBC (polyubiquitin) is an intriguing aspect of the host response, hinting at proteasomal involvement in RSV infection. One interpretation is that the host cell is responding to an increased need for protein degradation – perhaps due to misfolded viral proteins or the need to turnover signaling proteins after activation. Another compelling interpretation is that RSV actively manipulates the ubiquitin-proteasome system to antagonize host defenses. In fact, RSV encodes non-structural proteins NS1 and NS2 that are known to suppress type I interferon responses (Spann et al., 2004). Recent mechanistic studies have shown that RSV NS1 can form an E3 ubiquitin ligase complex by recruiting host factors, leading to targeted degradation of STAT2, a critical transcription factor in interferon signaling (Elliott et al., 2007). By degrading STAT2, RSV effectively blunts the interferon-mediated antiviral response. The dramatic increase in ubiquitin transcripts in our data could be a downstream consequence of NS1’s activity – as NS1 hijacks the cell’s ubiquitination machinery, the cell might upregulate ubiquitin expression to compensate. It is noteworthy that our array did not include interferon genes or many ISGs (interferon-stimulated genes), so we did not directly observe those in this experiment; however, NS1/NS2 function in interferon antagonism is well established (Spann et al., 2004; Elliott et al., 2007). Thus, the induction of UBC may serve as a marker of RSV’s immune evasion tactics at play. In essence, RSV prompts the host cell to increase ubiquitin availability, possibly to facilitate the degradation of host antiviral proteins. This hypothesis aligns with the idea that viruses often redirect host ubiquitin for their own purposes including RSV (Sedeyn et al., 2019)– a phenomenon observed in other viruses like influenza, whose NS1 targets ubiquitin ligases to disrupt RIG-I signaling (Koliopoulos et al., 2018). Our findings encourage further investigation into the ubiquitin pathways during RSV infection, as they could unveil targets for antiviral intervention (e.g., inhibiting specific E3 ligases to restore the interferon response).

Another key aspect of the host response to RSV highlighted by our results is the induction of inflammatory and immune mediators, particularly IL-6. IL-6 is a pleiotropic cytokine with both pro-inflammatory effects (fever, acute-phase protein induction, neutrophil recruitment) and regulatory roles (promoting antibody production and T-cell differentiation) (Luo and Zheng, 2016). The elevated IL-6 in RSV-infected HEp-2 cells is consistent with numerous *in vivo* studies where IL-6 is found in nasopharyngeal aspirates and bronchoalveolar fluid of children with RSV bronchiolitis (Russell et al., 2017). Clinically, higher IL-6 levels in airways or serum often correlate with more severe disease, such as the need for ventilation or greater hypoxemia (Russell et al., 2017) although IL-6 can also play a protective role by recruiting immune cells to clear infection. In our *in vitro* model, IL-6 production by epithelial cells likely reflects activation of innate sensing pathways (e.g., via toll-like receptors or RIG-I-like receptors recognizing RSV components) leading to NF-κB and AP-1 activation – transcription factors that drive IL-6 gene expression (Luo and Zheng, 2016). This occurs relatively early (within 24 h). The presence of IL-6 can have autocrine effects on the epithelial cells and paracrine effects on immune cells: for instance, IL-6 can enhance survival of lymphocytes and modulate the Th1/Th2 balance of T-cells in the context of RSV (Pyle et al., 2017). Some studies have proposed that IL-6 contributes to the mucus hypersecretion and airway hyperreactivity seen in RSV infections, by upregulating mucin genes and fostering a Th2-biased environment (Jafri et al., 2004). Conversely, IL-6 also promotes antiviral immune responses by stimulating acute phase reactions and B-cell antibody production (Castell et al., 1989; Maeda et al., 2010). Our observation of IL-6 upregulation confirms that even in isolated epithelial cells, RSV alone is sufficient to trigger a strong pro-inflammatory signal, which in an organism would amplify into the full syndrome of bronchiolitis. Managing the levels of such cytokines could be key to balancing viral clearance with immunopathology; hence, IL-6 could be a potential target for therapeutic modulation (for example, IL-6 blockers are used in other inflammatory diseases and might theoretically ameliorate severe RSV disease if given at the right time).

The induction of CD59 expression in RSV infection provides insight into how the virus may shield itself from the immune system. By increasing the availability of this complement inhibitor on cell surfaces, RSV-infected cells may better evade complement-mediated lysis. It has been documented that enveloped viruses can incorporate host cell membrane proteins as they bud; RSV budding occurs at the plasma membrane, so it can take along any host proteins present in those lipid rafts. If CD59 is enriched on infected cell membranes (due to gene upregulation and possible protein redistribution), new RSV virions can be cloaked in a complement-resistant coating. Favoreel et al. (2003) reviewed how viruses use such strategies to neutralize complement. Our data suggest RSV leverages this evasion mechanism by subtly upregulating a host gene rather than encoding its own mimic. This highlights an elegant aspect of viral strategy: co-opting host defense factors and turning them to the virus’s advantage. For the host, while CD59 protects normal cells from autologous complement attack, its increased presence on infected cells might unfortunately protect the virus as well. In the bigger picture, this exemplifies the arms race between host and pathogen – the host raises CD59 perhaps as a generic stress response to inflammation (to prevent “bystander” cell lysis), and the virus capitalizes on it. Therapeutically, this insight might inform approaches such as complement system modulation or designing vaccine strategies that account for such immune evasion.

Some of the limitations of the present study is the reliance on a single time point—24 hours post-infection (hpi). While this time point was selected to capture early transcriptional changes in response to RSV infection, gene expression is dynamic and evolves during the course of infection. Future time-course experiments at multiple intervals (e.g., 6, 12, 48, and 72 hpi) will be critical to determine the kinetics of host responses, including IFN signaling, cell cycle progression, and apoptosis. Incorporating temporal dynamics would improve the resolution of the molecular events driving RSV pathogenesis and allow us to distinguish between primary and secondary transcriptional effects.

Furthermore, our study utilized HEp-2 cells as a model of airway epithelial infection, which are highly permissive to RSV and suitable for transcriptional profiling. However, RSV-induced pathogenesis also involves interactions with immune cells including macrophages, dendritic cells, neutrophils, and lymphocytes, which were not assessed in this system. Future studies should consider co-culture models or *in vivo* systems that incorporate immune components to better mimic the host environment and to understand the immunological impact of RSV at the cellular interface.

Another critical next step involves functional validation of the identified host genes. Although we observed significant upregulation of genes such as CDKN1A (p21) and MCL1, their specific roles in RSV replication or host defense were not directly tested. Planned follow-up experiments include siRNA-mediated knockdown or pharmacological inhibition to assess the effect of gene silencing on viral replication, syncytium formation, and cell survival. Such mechanistic investigations will determine whether these genes represent host-protective responses or are exploited by RSV to facilitate replication.

In addition, our epithelial model does not fully capture RSV-induced immune dysregulation such as Th1/Th2 imbalance, regulatory T cell modulation, or immune-mediated tissue injury. Nonetheless, our observation of marked IL-6 upregulation may offer a clue into this dimension. IL-6 has been implicated in T cell differentiation, B cell activation, and mucin induction—all of which are central to RSV pathology. *In vivo* studies are required to evaluate how epithelial-derived IL-6 contributes to immunopathology and whether modulating this cytokine could represent a viable therapeutic strategy.

Collectively, our findings provide a robust transcriptomic framework for understanding epithelial responses to RSV. Despite some limitations, including single time point analysis and lack of immune cell inclusion or functional validation, this study lays the groundwork for future investigations aimed at dissecting gene function and host-pathogen interactions in RSV infection.

Finally, it is worth discussing the broader implications of these findings. The 12 genes highlighted by our microarray represent hubs of host response that could be experimentally interrogated for their role in RSV replication (**Figures 1**, **2**). For instance, does knocking down p21 in infected cells alter RSV yield? If p21 is crucial for optimizing replication (by arresting the cell cycle), one might see reduced viral production when p21 is absent, as the cells might enter apoptosis sooner or divert resources to proliferation. Conversely, if p21 is a host defense, its absence might let the virus spread unchecked. Similarly, blocking MCL1 (using inhibitors or siRNA) could test whether preventing the cell’s anti-apoptotic response leads to early cell death and limits RSV propagation. In the immunological context, neutralizing IL-6 or its receptor *in vivo* could determine how much of the pathology is IL-6 mediated – given IL-6’s complex role, this could potentially reduce harmful inflammation but might also impede efficient clearance. The upregulation of integrin α5 and RhoA suggests that drugs targeting RhoA signaling (like Rho-kinase inhibitors) might impact RSV’s ability to form syncytia; this could, for example, limit cell-cell spread of the virus in the lungs. Some of these hypotheses are supported by prior research (Pastey et al., 1999; Gower et al., 2005). Our transcriptomic data reinforce those findings at the gene expression level, and they provide a more comprehensive view by placing such observations alongside other host changes.

In conclusion, the host transcriptional program activated by RSV in epithelial cells encompasses defensive responses (cell cycle arrest, interferon pathway components indirectly indicated by ubiquitin, pro-inflammatory cytokines) as well as changes that likely facilitate viral replication (cytoskeletal reorganization, delayed apoptosis, complement regulators). RSV has evolved to tip the balance of these responses in its favor, at least transiently. By mapping these changes, our study contributes to a deeper understanding of RSV pathogenesis. The molecular snapshot obtained here generates multiple testable hypotheses about virus-host interactions. Future work can extend these findings by examining time-course expression (to see early interferon responses or late-stage cell death genes), by investigating protein-level changes corresponding to these mRNA changes, and by using reverse genetics on RSV or host gene knockouts to validate the functional importance of each identified host factor. Ultimately, insights from such studies may guide the development of novel antiviral strategies – for example, targeting host factors like those identified here could be an approach to circumvent issues of antiviral resistance. As RSV continues to be a major pediatric pathogen without a licensed vaccine for infants (as of this writing), research into these host pathways not only expands our knowledge of virus biology but could also pave the way for host-directed therapies or improved clinical management of severe RSV disease.

## Data Availability

The data generated and analyzed during the current study are available from the corresponding author upon reasonable request. This study does not include mandatory data types requiring deposition in a public repository, and all relevant data are either included within the article or available upon request in accordance with Frontiers' open data policies.

## References

[B1] Agrawal P. Nawadkar R. Ojha H. Kumar J. Sahu A. (2017). Complement evasion strategies of viruses: an overview. Front. Microbiol. 8. doi: 10.3389/fmicb.2017.01117 PMC5472698 28670306

[B2] Ampuero S. Andaur R. Milano M. Moreno M. Lizama L. Larrañaga C.. (2018). Time-course of transcriptome response to respiratory syncytial virus infection in lung epithelium cells. Acta Virologica. 62, 310–325. doi: 10.4149/av_2018_225 30160147

[B3] Arrindell J. Desnues B. (2023). Vimentin: from a cytoskeletal protein to a critical modulator of immune response and a target for infection. Front. Immunol. 14. doi: 10.3389/fimmu.2023.1224352 PMC10354447 37475865

[B4] Bachmann M. Kukkurainen S. Hytönen V. P. Wehrle-Haller B. (2019). Cell adhesion by integrins. Physiol. Rev. 99, 1655–1699. doi: 10.1152/physrev.00036.2018 31313981

[B5] Bedient L. Pokharel S. M. Chiok K. R. Mohanty I. Beach S. S. Miura T. A.. (2020). Lytic cell death mechanisms in human respiratory syncytial virus-infected macrophages: roles of pyroptosis and necroptosis. Viruses 12, 932. doi: 10.3390/v12090932 32854254 PMC7552060

[B6] Brown G. Jeffree C. E. McDonald T. Rixon H. W. Aitken J. D. Sugrue R. J. (2004). Analysis of the interaction between respiratory syncytial virus and lipid-rafts in HEp-2 cells during infection. Virology 327, 175–185. doi: 10.1016/j.virol.2004.06.038 15351205

[B7] Castell J. V. Gómez-Lechón M. J. David M. Andus T. Geiger T. Trullenque R.. (1989). Interleukin-6 is the major regulator of acute phase protein synthesis in adult human hepatocytes. FEBS Lett. 242, 237–239. doi: 10.1016/0014-5793(89)80476-4 2464504

[B8] Danen E. H. Sonneveld P. Brakebusch C. Fassler R. Sonnenberg A. (2002). The fibronectin-binding integrins alpha5beta1 and alphavbeta3 differentially modulate RhoA-GTP loading, organization of cell matrix adhesions, and fibronectin fibrillogenesis. J. Cell Biol. 159, 1071–1086. doi: 10.1083/jcb.200205014 12486108 PMC2173988

[B9] Elder R. T. Yu M. Chen M. Edelson S. Zhao Y. (2000). Cell cycle G2 arrest induced by HIV-1 Vpr in fission yeast (Schizosaccharomyces pombe) is independent of cell death and early genes in the DNA damage checkpoint. Virus Res. 68, 161–173. doi: 10.1016/s0168-1702(00)00167-2 10958988

[B10] Elliott J. Lynch O. T. Suessmuth Y. Qian P. Boyd C. R. Burrows J. F.. (2007). Respiratory syncytial virus NS1 protein degrades STAT2 by using the elongin-cullin E3 ligase. J. Virol. 81 (7), 3428–3436. doi: 10.1128/jvi.02303-06 PMC1866062 17251292

[B11] Favoreel H. W. Van de Walle G. R. Nauwynck H. J. Pensaert M. B. (2003). Virus complement evasion strategies. J. Gen. Virol. 84, 1–15. doi: 10.1099/vir.0.18709-0 12533696

[B12] Garcia-Barreno B. Jorcano J. L. Aukenbauer T. López-Galíndez C. Melero J. A. (1988). Participation of cytoskeletal intermediate filaments in the infectious cycle of human respiratory syncytial virus (RSV). Virus Res. 9, 307–321. doi: 10.1016/0168-1702(88)90090-1 2837016

[B13] Goh W. C. Rogel M. E. Kinsey C. M. Michael S. F. Fultz P. N. Nowak M. A.. (1998). HIV-1 Vpr increases viral expression by manipulation of the cell cycle: a mechanism for selection of Vpr *in vivo* . Nat. Med. 4, 65–71. doi: 10.1038/nm0198-65 9427608

[B14] Gower T. L. Pastey M. K. Peeples M. E. Collins P. L. McCurdy L. H. Hart T. K.. (2005). RhoA signaling is required for respiratory syncytial virus-induced syncytium formation and filamentous virion morphology. J. Virol. 79, 5326–5336. doi: 10.1128/JVI.79.9.5326-5336.2005 15827147 PMC1082718

[B15] Granville D. J. Carthy C. M. Yang D. Hunt D. W. McManus B. M. (1998). Interaction of viral proteins with host cell death machinery. Cell Death differentiation 5, 653–659. doi: 10.1038/sj.cdd.4400388 10200520

[B16] Hosakote Y. M. Liu T. Castro S. M. Garofalo R. P. Casola A. (2009). Respiratory syncytial virus induces oxidative stress by modulating antioxidant enzymes. Am. J. Resp. Cell Mol. Biol. 41 (3), 348–357. doi: 10.1165/rcmb.2008-0330OC PMC2742754 19151318

[B17] Jafri H. S. Chavez-Bueno S. Mejias A. Gomez A. M. Rios A. M. Nassi S. S.. (2004). Respiratory syncytial virus induces pneumonia, cytokine response, airway obstruction, and chronic inflammatory infiltrates associated with long-term airway hyperresponsiveness in mice. J. Infect. Dis. 189, 1856–1865. doi: 10.1086/386372 15122522

[B18] Koliopoulos M. G. Lethier M. van der Veen A. G. Haubrich K. Hennig J. Kowalinski E.. (2018). Molecular mechanism of influenza A NS1-mediated TRIM25 recognition and inhibition. Nat. Commun. 9, 1820. doi: 10.1038/s41467-018-04214-8 29739942 PMC5940772

[B19] Koval C. E. Gonzalez B. E. (2024). RSV in transplant and immunocompromised patients. Cleveland Clinic J. Med. 91, S34–S41. doi: 10.3949/ccjm.91.s1.06 39231600

[B20] Levitz R. Wattier R. Phillips P. Solomon A. Lawler J. Lawler I.. (2012). Induction of IL-6 and CCL5 (RANTES) in human respiratory epithelial (A549) cells by clinical isolates of respiratory syncytial virus is strain specific. Virol. J. 9, 190. doi: 10.1186/1743-422X-9-190 22962966 PMC3463437

[B21] Luo Y. Zheng S. G. (2016). Hall of fame among pro-inflammatory cytokines: interleukin-6 gene and its transcriptional regulation mechanisms. Front. Immunol. 7. doi: 10.3389/fimmu.2016.00604 PMC5165036 28066415

[B22] Maeda K. Mehta H. Drevets D. A. Coggeshall K. M. (2010). IL-6 increases B-cell IgG production in a feed-forward proinflammatory mechanism to skew hematopoiesis and elevate myeloid production. Blood 115, 4699–4706. doi: 10.1182/blood-2009-07-230631 20351305 PMC3790945

[B23] Martínez I. Lombardía L. García-Barreno B. Domínguez O. Melero J. A. (2007). Distinct gene subsets are induced at different time points after human respiratory syncytial virus infection of A549 cells. J. Gen. Virol. 88, 570–581. doi: 10.1099/vir.0.82187-0 17251576

[B24] Mbita Z. Hull R. Dlamini Z. (2014). Human immunodeficiency virus-1 (HIV-1)-mediated apoptosis: new therapeutic targets. Viruses 6, 3181–3227. doi: 10.3390/v6083181 25196285 PMC4147692

[B25] Morrison P. T. Thomas L. H. Sharland M. Friedland J. S. (2007). RSV-infected airway epithelial cells cause biphasic up-regulation of CCR1 on human monocytes via secreted cytokines. J. Leukoc. Biol. 81, 1487–1495. doi: 10.1189/jlb.1006611 17389578

[B26] Nair H. Nokes D. J. Gessner B. D. Dherani M. Madhi S. A. Singleton R. J.. (2010). Global burden of acute lower respiratory infections due to respiratory syncytial virus in young children: a systematic review and meta-analysis. Lancet 375, 1545–1555. doi: 10.1016/S0140-6736(10)60206-1 20399493 PMC2864404

[B27] Noah T. L. Henderson F. W. Wortman I. A. Devlin R. B. Handy J. Koren H. S.. (1995). Nasal cytokine production in viral acute upper respiratory infection of childhood. J. Infect. Dis. 171, 584–592. doi: 10.1093/infdis/171.3.584 7876605

[B28] Oshansky C. M. Zhang W. Moore E. Tripp R. A. (2009). The host response and molecular pathogenesis associated with respiratory syncytial virus infection. Future Microbiol. 4, 279–297. doi: 10.2217/fmb.09.1 19327115 PMC2711508

[B29] Pastey M. K. Crowe J. E. Jr. Graham B. S. (1999). RhoA interacts with the fusion glycoprotein of respiratory syncytial virus and facilitates virus-induced syncytium formation. J. Virol. 73, 7262–7270. doi: 10.1128/JVI.73.9.7262-7270.1999 10438814 PMC104251

[B30] Pyle C. J. Uwadiae F. I. Swieboda D. P. Harker J. A. (2017). Early IL-6 signalling promotes IL-27 dependent maturation of regulatory T cells in the lungs and resolution of viral immunopathology. PloS Pathog. 13, e1006640. doi: 10.1371/journal.ppat.1006640 28953978 PMC5633202

[B31] Russell C. D. Unger S. A. Walton M. Schwarze J. (2017). The human immune response to respiratory syncytial virus infection. Clin. Microbiol. Rev. 30 (2), 481–502. doi: 10.1128/cmr.00090-16 PMC5355638 28179378

[B32] Sedeyn K. Schepens B. Saelens X. (2019). Respiratory syncytial virus nonstructural proteins 1 and 2: Exceptional disrupters of innate immune responses. PloS Pathog. 15, e1007984. doi: 10.1371/journal.ppat.1007984 31622448 PMC6797084

[B33] Sigurs N. Bjarnason R. Sigurbergsson F. Kjellman B. (2000). Respiratory syncytial virus bronchiolitis in infancy is an important risk factor for asthma and allergy at age 7. Am. J. Respir. Crit. Care Med. 161, 1501–1507. doi: 10.1164/ajrccm.161.5.9906076 10806145

[B34] Smith J. A. (2018). Regulation of cytokine production by the unfolded protein response; implications for infection and autoimmunity. Front. Immunol. 9. doi: 10.3389/fimmu.2018.00422 PMC5844972 29556237

[B35] Spann K. M. Tran K. Chi B. Rabin R. L. Collins P. L. (2004). Suppression of the induction of alpha, beta, and gamma interferons by the NS1 and NS2 proteins of human respiratory syncytial virus in human epithelial cells and macrophages. J. Virol. 78 (8), 4363–4369. doi: 10.1128/jvi.78.8.4363-4369.2004 PMC374276 15047850

[B36] Stewart S. A. Poon B. Song J. Y. Chen I. S. (2000). Human immunodeficiency virus type 1 vpr induces apoptosis through caspase activation. J. Virol. 74, 3105–3111. doi: 10.1128/jvi.74.7.3105-3111.2000 10708425 PMC111809

[B37] Surolia R. Antony V. B. (2022). Pathophysiological role of vimentin intermediate filaments in lung diseases. Front. Cell Dev. Biol. 10. doi: 10.3389/fcell.2022.872759 PMC9096236 35573702

[B38] Tian B. Zhang Y. Luxon B. A. Garofalo R. P. Casola A. Sinha M.. (2002). Identification of NF-kappaB-dependent gene networks in respiratory syncytial virus-infected cells. J. Virol. 76, 6800–6814. doi: 10.1128/jvi.76.13.6800-6814.2002 12050393 PMC136270

[B39] Townsend D. M. Tew K. D. (2003). The role of glutathione-S-transferase in anti-cancer drug resistance. Oncogene 22, 7369–7375. doi: 10.1038/sj.onc.1206940 14576844 PMC6361125

[B40] Whimbey E. Couch R. B. Englund J. A. Andreeff M. Goodrich J. M. Raad I. I.. (1995). Respiratory syncytial virus pneumonia in hospitalized adult patients with leukemia. Clin. Infect. Dis. 21, 376–379. doi: 10.1093/clinids/21.2.376 8562747

[B41] WHO. (2025). Available online at: https://www.who.int/news-room/fact-sheets/detail/respiratory-syncytial-virus-(rsv):~:text=%28WHO%29%20www (Accessed March 5, 2025).

[B42] Yang X. Liu X. Nie Y. Zhan F. Zhu B. (2023). Oxidative stress and ROS-mediated cellular events in RSV infection: potential protective roles of antioxidants. Virol. J. 20, 224. doi: 10.1186/s12985-023-02194-w 37798799 PMC10557227

[B43] Zhang Y. Luxon B. A. Casola A. Garofalo R. P. Jamaluddin M. Brasier A. R. (2001). Expression of respiratory syncytial virus-induced chemokine gene networks in lower airway epithelial cells revealed by cDNA microarrays. J. Virol. 75, 9044–9058. doi: 10.1128/JVI.75.19.9044-9058.2001 11533168 PMC114473

